# sciCAN: single-cell chromatin accessibility and gene expression data integration via cycle-consistent adversarial network

**DOI:** 10.1038/s41540-022-00245-6

**Published:** 2022-09-12

**Authors:** Yang Xu, Edmon Begoli, Rachel Patton McCord

**Affiliations:** 1 UT-ORNL Graduate School of Genome Science and Technology, University of Tennessee, Knoxville, TN USA; 2 Oak Ridge National Laboratory, Oak Ridge, TN USA; 3 Electrical Engineering and Computer Science, University of Tennessee, Knoxville, TN USA; 4 Biochemistry & Cellular and Molecular Biology Department, University of Tennessee, Knoxville, TN USA

**Keywords:** Software, Molecular biology

## Abstract

The boom in single-cell technologies has brought a surge of high dimensional data that come from different sources and represent cellular systems from different views. With advances in these single-cell technologies, integrating single-cell data across modalities arises as a new computational challenge. Here, we present an adversarial approach, sciCAN, to integrate single-cell chromatin accessibility and gene expression data in an unsupervised manner. We benchmarked sciCAN with 5 existing methods in 5 scATAC-seq/scRNA-seq datasets, and we demonstrated that our method dealt with data integration with consistent performance across datasets and better balance of mutual transferring between modalities than the other 5 existing methods. We further applied sciCAN to 10X Multiome data and confirmed that the integrated representation preserves biological relationships within the hematopoietic hierarchy. Finally, we investigated CRISPR-perturbed single-cell K562 ATAC-seq and RNA-seq data to identify cells with related responses to different perturbations in these different modalities.

## Introduction

Within the last decade, single-cell technologies have advanced our understanding in a broad range of biological systems. Single-cell RNA-seq and single-cell ATAC-seq, along with other single-cell assays, have revealed distinct cellular heterogeneity at a comprehensive level, from genomic variations to epigenomic modifications and transcriptomic regulation^1–5^. Analyses based on single-cell data have also provided reliable databases for biomedical research and valuable references for medical discovery. As the number of single-cell omics datasets grows, there is increasing demand for fast and accurate computation. Consequently, deep learning has become a trending topic in single-cell data analysis. Much recent research has focused on developing reliable and fast deep learning tools to accommodate the scaling demand, such as cell-type annotation^6^, doublet identification^7^, data de-noising^8^, and batch correction^9^.

Among all applications of deep learning in single-cell analysis, data integration remains one of the grand challenges in the community^10,11^. Multiple single-cell RNA-seq platforms were simultaneously developed, leading to an initial focus on methods to integrate datasets from different platforms. Batch effects are usually the most prominent variation when datasets from different sources are collected for integrative analysis, and this can obscure meaningful biological information. Therefore, removing batch effects is a critical step to reveal true biological variation and is necessary for building batch-invariant and applicable single cell atlases. So far, multiple methods have been proposed to address this problem^9,12–16^. Among these integration methods, deep generative models were extensively tested and demonstrated their efficacy for learning discriminative representations from the original high dimensional space. The most common generative models are Variational Autoencoder (VAE). Variants of VAE models, which differ in their sampling approaches, have been proposed to learn representations for single-cell gene expression data^9,17–20^. The core component of VAE is the use of reconstruction loss, which encodes a sample in a representation that is drawn from a certain distribution, for example, a Gaussian distribution. The use of reconstruction loss also has an advantage of mapping noisy data to high-quality data, which further extends the ability of generative model to de-noise data or impute gene expression. Instead of using VAE to learn representation for single-cell RNA-seq data, two research groups simultaneously modified VAE to address batch effects using an adversarial approach^19,20^. These two methods, named scGAN and AD-AE, respectively, used generative adversarial network (GAN) as the main framework for learning the latent space that is not entangled with batch effects. Starting from a VAE model, both scGAN^19^ and AD-AE^20^ introduced adversarial domain loss into the generative model and transferred the learning from reconstruction of data to diminishing of non-biological variation. This approach turned out to be effective in removing batch effects within single-cell gene expression data.

However, both scGAN and AD-AE solely focused on the use of adversarial learning in single-cell RNA-seq data. Considering their success in batch-effect correction, here, we aim to extend the use of adversarial learning to single-cell data integration across different modalities. In this study, we focus on modality differences and develop an improved adversarial domain adaptation approach to address multimodal data integration for chromatin accessibility (ATAC-seq) and gene expression (RNA-seq) data. Our method differs from both scGAN and AD-AE in that it uses a cycle-consistent adversarial network to learn the joint representation for both chromatin accessibility and gene expression data^21^. We term our method sciCAN (single-cell chromatin accessibility and gene expression data ***i***ntegration via Cycle-consistent Adversarial Network), which removes modality differences while keeping true biological variation. We previously developed a deep learning method, SMILE, to perform integration of multimodal single-cell data^22^. SMILE requires cell anchors for integration. This limits the use of SMILE only to cases where corresponding cells are known across modalities, as is true for joint profiling experiments. In contrast to SMILE, sciCAN does not require cell anchors and thus can be applied to non-joint profiled single-cell data. We first benchmarked our method with 5 existing methods across 5 ATAC-seq/RNA-seq datasets, and we demonstrate that our method deals with data integration with a better ability to transfer cell type labels in both directions between modalities than the other 5 methods. To demonstrate the method’s utility in integrative analyses, we applied sciCAN to joint-profiled peripheral blood mononuclear cells (PBMC) data by 10X Multiome platform and showed that the derived integrated representation preserves the hematopoietic hierarchy at both chromatin accessibility and gene expression levels. Finally, we investigated CRSIPR-perturbed single-cell K562 ATAC-seq and RNA-seq data, and we identified that some cells in both modalities share common biological responses, even though the two modalities were profiled with different gene perturbations. Combining the results above, we expect our work will fill the gap to allow generative models to be used in integrative analysis of multimodal single-cell data.

## Results

### Overview of sciCAN and potential applications

**Fig. 1:**
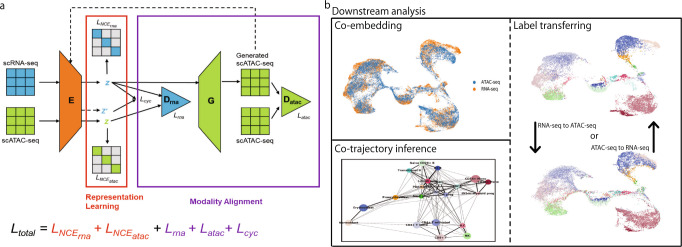
Overview of sciCAN and potential applications. **a** sciCAN model architecture. sciCAN contains two major components: representation learning and modality alignment. The representation learning part of the model is highlighted in the red box, and the modality alignment part in the purple box. Inputs of scATAC-seq and scRNA-seq have been preprocessed to have the same feature dimensions, so they can share one single encoder *E*. The final total loss (*L*) is the sum of loss of representation learning in red and loss of modality alignment in purple. Of note, calculation of *NCE* is independent for scATAC-seq and scRNA-seq data. **b** Downstream integrative analyses can include but are not limited to co-embedding, co-trajectory, and label transferring.

We first show the model architecture of sciCAN, which contains two major components, representation learning and modality alignment (Fig. 1a). Encoder *E* serves as a feature extractor that projects both high dimensional chromatin accessibility and gene expression data into the joint low dimension space. For representation learning, we use noise contrastive estimation (NCE) as the single loss function to guide *E* to learn the discriminative representation that can preserve the intrinsic data structure for both modalities. For modality alignment, we use two separate discriminator networks for two distinct uses. The first discriminator network *D*_*rna*_ is attached to *E* and is trained with adversarial domain adaptation loss. *D*_*rna*_ aims to distinguish which source the latent space *z* extracted by *E* comes from, while *E* is pushed to learn the joint distribution so that *D*_*rna*_ is less able to distinguish the modality source of latent space *z*. The second discriminator network *D*_*atac*_ follows a generator network *G* that generates chromatin accessibility data based latent space *z* from gene expression data. Adversarial training here will push *G* to find a connection between chromatin accessibility and gene expression data. Since the generated chromatin accessibility data is based on the latent space *z* of real gene expression data, the new latent space *z’* of generated data should align with its corresponding *z* of real gene expression data. Therefore, we add cycle-consistent loss as demonstrated in cycleGAN method to facilitate finding the connection between two modalities^21^. In practice, we build *E* with fully connected layers, which are followed by a batch normalization layer with Rectified Linear Unit (ReLU) activation. *D*_*rna*_ takes the 128-dimension *z* as input and forwards it through a three-layer multi-layer perceptron (MLP) to produce 1-dimension sigmoid activated output that predicts if the input *z* comes from single-cell RNA-seq data. Differently, *D*_*atac*_ takes output from *G* and forwards the input through a three-layer MLP to produce 1-dimension sigmoid activated output that predicts if input is generated by *G*. *G* is a decoder structure, which has two-layer MLP to restore dimension-reduced *z* to the original dimension of input data. Instead of calculating NCE directly on *z*, we further reduced *z* to 32-dimension output with linear transformation and 25-dimension SoftMax activated output, through two separated one-layer MLPs. This practice is the same as our previous study, in which we demonstrated an effective approach to learn discriminative representation for single-cell data^22^. Once model training is done, we use encoder *E* to project both modalities into the joint representation for downstream analyses (Fig. 1b).

### Benchmark of sciCAN with 5 existing integration methods

To demonstrate the performance of sciCAN in the task of data integration, we first selected 3 methods for comparison that have been extensively tested in integrating single-cell RNA-seq data^23^, including LIGER^14^, Harmony^15^, and Seurat^24^. In addition to these specialized methods for integration, we noticed the availability of streamlined analysis tools for single-cell ATAC-seq data, including ArchR^25^, MAESTRO^26^, and Cicero^27^. Of these, both ArchR and MAESTRO include the ability to integrate ATAC-seq and RNA-seq data, using Seurat directly (MAESTRO) or with further modifications to Seurat (ArchR). Thus, we included ArchR in our benchmark test. As sciCAN shares the same architecture as SMILE to learn representations for single-cell data and both methods are proposed for data integration, we also included SMILE in our set of methods to test. However, SMILE requires cell anchors across modalities to learn the joint representation, while not all benchmark datasets include this information. Therefore, we used Seurat to identify cell anchors so that SMILE could rely on these anchors to integrate ATAC-seq and RNA-seq data. For the benchmark purpose, we collected 5 datasets that consist of distinct cellular systems. They are mixed cell lines^28^, human hematopoiesis^29^, human lung^30^, mouse skin^31^, and mouse kidney^32^, respectively. RNA-seq and ATAC-seq modalities may have different numbers of cells and even different numbers of cell types, except where both modalities were jointly profiled (Supplementary Table 1).

**Fig. 2:**
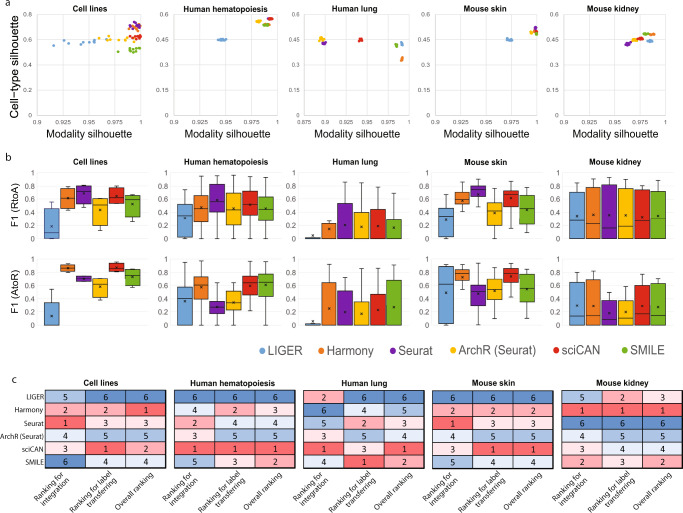
Benchmarking of sciCAN against other 5 existing integration methods. **a** Integration evaluation by modality and cell-type silhouette scores across 5 datasets. *x* axis corresponds to modality silhouette score and *y* axis to cell-type silhouette score. Ideal integration should be located in the top right corner of each dot plot. To generate the dot plot, we randomly subsample 20% of the cell population to calculate both modality and cell-type silhouette scores for each method and each dataset. **b** Integration evaluation by F1 scores across 5 datasets. Upper panel corresponds to label transferring from RNA-seq to ATAC-seq (RtoA) while lower panel indicates label transferring from ATAC-seq to RNA-seq (AtoR). Boxplots are plotted based on F1 scores for all cell types in that dataset. The median value is marked with a horizontal line within the box, and the “X” mark represents the macro F1 score, which is the average of F1 scores for all cell types. Whiskers show minimum and maximum value and top and bottom of the box show 25th and 75th percentile respectively. **c** Benchmark ranking across 5 datasets. In each category, methods are ranked based on their scores from best (red, low number ranking) to worst (blue, high number ranking).

We introduced two variants of silhouette score to measure modality mixing and cell-type preserving, respectively. The first metric, modality silhouette, evaluates how well two modalities align, and it directly reports whether discrepancy between chromatin accessibility and gene expression data is removed (maximum alignment gives a score of 1). Across 5 datasets, Harmony, Seurat, and sciCAN integrated chromatin accessibility and gene expression data well, giving a larger modality silhouette value. Among all methods, LIGER ranked the last in modality mixing, with the worst modality silhouette values in 3 datasets (Fig. 2a). Though all 6 methods diminish the difference between chromatin accessibility and gene expression, that does not necessarily indicate that they learned to represent the distinctness of each cell-type. To evaluate this, we used cell-type silhouette, which quantifies how well the joint representation reflects the data structure by distinguishing cell-types (a value of 1 is ideal). Here, we used the author-reported labels as the ground truth. All other 5 methods, except sciCAN, reported the last-ranked cell-type silhouette in the 5 datasets at least once (Fig. 2a). Though ArchR performs integration upon infrastructure of Seurat, we observed noticeable difference between ArchR and Seurat. Different from Seurat that maps connections between RNA-seq and ATAC-seq data as whole, ArchR only does the “subspace” mapping^25^, and this “subspace” mapping is highly influenced by a good estimation on correspondence between RNA-seq “subspace” and ATAC-seq “subspace”. Considering the goal of a good balance between modality mixing and cell-type preserving, sciCAN shows the most consistent integration performance across the 5 datasets among all methods.

Next, we focused on label transferring. Here, our goal is that the user could rely on the integrated space to predict cell-type labels for data from a single modality, given availability of cell-type labels from the other modality. We found Seurat has overall the best performance for label transferring from RNA-seq to ATAC-seq (Fig. 2b). This may relate to the design of Seurat. Different from the other 3 methods, Seurat inherently uses gene expression data as reference data and projects chromatin accessibility data to the gene expression space. In contrast, sciCAN has overall the best performance of label transferring from ATAC-seq to RNA-seq (Fig. 2b). Among all methods, LIGER shows the worst performance regarding label transferring (Fig. 2b). Visual inspection of the integration also indicates that Harmony, Seurat, and sciCAN show overall better integration performance than the other 3 methods (Supplementary Figs. 1–5). Finally, we ranked these 6 integration methods in both tasks of data integration and label transferring and got the ranking for overall performance (Fig. 2c). In 4 out of 5 datasets, sciCAN was ranked in the top 3 methods in both tasks of data integration and label transferring. sciCAN was ranked highest overall 3 times and Harmony 2 times. A statistical comparison of benchmarking metrics confirmed that while sciCAN does not always significantly outperform other methods on all metrics, it has the best consistent performance in label transferring between modalities, particularly when starting from ATAC-seq data. (Supplementary Table 2).

The default architecture of sciCAN shown in Fig. 1 has RNA-seq data playing the central role, primarily because RNA-seq data usually shows greater discriminative power than ATAC-seq in terms of cell-type identification^22,24,33–35^. We wondered if this setup is critical to good integration by sciCAN. Thus, we switched the roles of RNA-seq and ATAC-seq data in the model training. Indeed, the ATAC-centered sciCAN model is consistently less accurate than RNA-centered sciCAN, suggesting discriminative representation learning benefits from taking advantage of the cell-type discriminative power of RNA-seq (Supplementary Fig. 6). We also evaluated the contribution of each loss in the final sciCAN model in two joint-profiled datasets of different size and complexity. We found that the different coefficients of the loss functions do not have major impact on model performance. However, larger coefficients for *L*_*atac*_ returned the worst outcomes compared to other loss coefficient sets we tested (Supplementary Table 3). This evaluation further supports the idea that taking advantage of the discriminative power of scRNA-seq returns better integration. Combining the results above, we conclude that the RNA-centered sciCAN shows consistently good integration performance across different cellular systems.

### Integration learned by sciCAN preserves the hematopoietic hierarchy

**Fig. 3:**
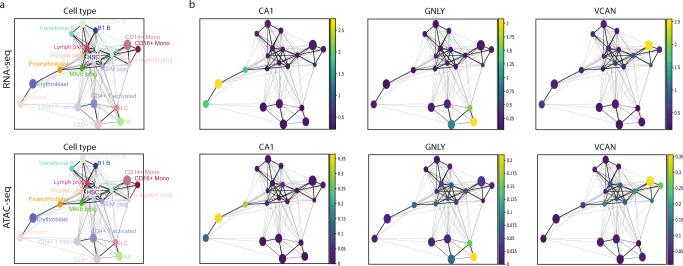
Integration learned by sciCAN preserves hematopoietic hierarchy. **a** Co-trajectory analysis via PAGA using joint representation learned by sciCAN. Each dot is the sum of all cells annotated as the same cell type. Trajectory is visualized using RNA-seq (upper panel) and ATAC-seq (lower panel), separately. **b** Enrichments of signature genes for 3 different lineages using both RNA-seq (top) and ATAC-seq (bottom) data. Colorbar indicates gene expression (top) or gene activity level (bottom), respectively.

The hematopoietic hierarchy has been extensively studied through single-cell analysis. Independent studies using scRNA-seq or scATAC-seq alone also confirmed that the cellular hierarchy of the hematopoietic system is observed at both chromatin accessibility and gene expression levels^36–40^. Thus, hematopoietic data can be a good example for us to verify whether the learned integration is biologically meaningful. In our benchmark above, all 6 methods showed decent integration for human hematopoiesis. However, when we inspected how well the learned integration preserved known cellular relationships, we found that only LIGER, sciCAN and SMILE highlighted distinct groups of biologically related cell types, while the other 3 methods returned weak correlations and showed some closely related cell types as distantly separated (Supplementary Fig. 7). To demonstrate that sciCAN indeed learns biologically meaningful integration, we performed further investigation into the hematopoietic hierarchy. Instead of using scRNA-seq and scATAC-seq data that were profiled separately, as the dataset we used in the benchmark, we utilized a jointly-profiled human PBMC dataset obtained through the 10X Multiome platform, which enables us to evaluate the integration with ground truth. Blinding ourselves to cell pairing information, our first task is co-embedding RNA-seq and ATAC-seq and performing co-trajectory analysis to evaluate whether the joint representation learned by sciCAN preserves the hematopoietic hierarchy at both chromatin accessibility and gene expression levels. Indeed, PAGA, a trajectory inference tool for single-cell data, constructed a hematopoietic stem cell (HSC)-centered trajectory with the 128-dimension joint representation learned by sciCAN^41^. We also confirmed that progenitor cells surround the HSCs and branch towards their differentiated cells, and their lineage commitments at both chromatin accessibility and gene expression levels can be explained by the same gene signatures (Fig. 3b and Supplementary Fig. 8).

Given that the integrated representation learned by sciCAN preserved the hematopoietic hierarchy, we next asked if we could infer transcriptional dynamics between chromatin accessibility and gene expression across the trajectory from progenitor to differentiated cells. To do so, we borrowed and transformed the concept of RNA velocity into activity-expression velocity. In the original RNA velocity concept, positive velocity is inferred when an increase in unspliced transcripts is followed by up-regulation in spliced transcripts^42,43^. This idea was further extended to velocity analysis of nuclear mRNA vs cytoplasmic mRNA^44^, and of more compact vs less compact chromatin regions^45^. Here, we reframed this analysis into activity-expression velocity. We found that the trajectories of the resulting velocity calculation follow the expected hematopoietic differentiation (from stem and progenitor to differentiated type) when we calculate positive velocity as an increase in gene expression first, followed by an increase in gene activity (accessibility). This directionality suggests that in this system gene expression may be activated first, followed by a chromatin state encoding of this expression pattern as the new cell type is established. Given the joint representation, we predicted gene expression based on gene activity. Then, we used the true activity matrix and the predicted expression matrix to compute the activity-expression velocity with scVelo^46^. Taking advantage of the ground truth from the cell pairing information, we also performed the same analysis using the true activity matrix and true expression matrix. We found that velocity computed with the predicted expression data resembles and correlates well with the velocity computed with true expression data, in accordance with the correlation between predicted and actual expression (Supplementary Fig. 9). Consistent with co-trajectory analysis, velocity with predicted expression data revealed that MK/E progenitor cells move towards erythroblasts while G/M progenitor cells move towards monocytes (Supplementary Fig. 9). Combining the results above, we concluded that sciCAN preserves meaningful biological information within the learned joint representation.

### sciCAN identifies common responses after CRISPR perturbation

**Fig. 4:**
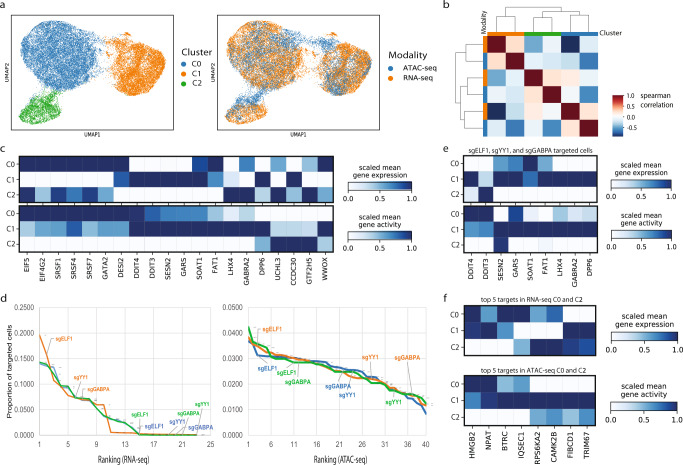
sciCAN identifies common response after CRISPR perturbation. **a** Visualization of single-cell CRISPR-perturbed K562 RNA-seq and ATAC-seq data via UMAP. Cells are colored by identified cell clusters (left) and modality source (right). **b** Spearman correlation between RNA-seq and ATAC-seq profiles of cells in different clusters in both modalities. Gene expression or gene activity matrix was averaged by cell clusters. **c** Shared gene signatures of the 3 cell clusters in both modalities. Differential gene activities or expression were identified through ‘Wilcoxon’ test in Scanpy package. **d** Ranking of sgRNA representation in each cluster (blue = C0, orange = C1, green = C2) in both RNA-seq (left) and ATAC-seq (right) data. Genes perturbed in both experiments are highlighted. **e** Gene signatures of cells targeted by *sgELE1*, *sgYY1*, and *sgGABPA* in cell cluster 1. **f** Genes whose activity patterns distinguish cells in cluster 0 and cluster 2 among cells in these clusters perturbed by the same gRNAs.

Combining single-cell sequencing with CRISPR enables a systematic examination of cellular response to genetic perturbation. Dixit et al. first introduced Perturb-seq to identify single-cell cellular response at the expression level after CRISPR perturbation^47^. Then, Perturb-ATAC was introduced to profile single-cell chromatin accessibility after CRISPR perturbation^48^. Nevertheless, a CRISPR-coupled joint-profiling single-cell assay has not been introduced. Therefore, multiple modality data integration is needed to determine how single cell responses to genetic perturbation compare at the transcriptomic and chromatin accessibility levels. We performed computational integration via sciCAN to create a joint view of cellular response after CRISPR perturbation. We selected single-cell K562 RNA-seq data from Perturb-seq and single-cell K562 ATAC-seq data generated by Spear-ATAC^47,49^. Notably these two studies used quite different sgRNA sets, sharing only 3 gene targets (*sgELF1*, *sgYY1*, and *sgGABPA*), so the integration cannot simply group like targets, but instead will be challenged to find similar biological responses to different gene perturbations. First, sciCAN enabled us to co-embed and co-cluster RNA-seq and ATAC-seq data, and we identified 3 distinct clusters (Fig. 4a). Next, we asked if the co-clustering makes sense in terms of gene signatures that lead to these clusters. Though the two studies used different sgRNA sets, we found gene activities of these 3 clusters have strong correlation to the gene expression profiles of the corresponding clusters in RNA-seq (Fig. 4b). This correlation surpasses the cellular correlation calculated with 5 benchmark datasets in which known cell types are present in both ATAC-seq and RNA-seq modalities (Supplementary Fig. 10). Further, cells within each cluster shared gene signatures in both expression and accessibility (Fig. 4c). This suggests that cells may have similar responses to different CRISPR-perturbations. Next, we ranked sgRNA targets for each cluster to find out which genes were perturbed in the cells that ended up in each cluster. In RNA-seq data, cells targeted by sgELF1, sgYY1, and sgGABPA tended to fall in cluster 1 (Fig. 4d). However, even though these three genes were also targeted in ATAC-seq, the cells targeted by these sgRNAs were fairly evenly distributed across clusters in scATAC-seq data (Fig. 4d and Supplementary Table 4). We reason that cellular responses to perturbation at the chromatin accessibility level may be more variable than the responses at the gene expression level. Indeed, none of the ATAC-seq cell clusters have strongly dominant sgRNA targets (Supplementary Table 4). To further investigate this discrepancy, we separated out cells that were targeted by the common targets *sgELF1*, *sgYY1*, and *sgGABPA* for a closer examination. We found that cells targeted by *sgELF1*, *sgYY1*, and *sgGABPA* that fall into cluster 1 in both RNA-seq and ATAC-seq do have a distinct gene expression and activity signature compared to cluster 0 and 2, even though these cells were perturbed by the same sgRNAs (Fig. 4e). This suggests that there are different subsets of cells that respond to the same perturbation in different ways. Shifting our focus to clusters 0 and 2 overall, it is surprising that cells in these two clusters share the same top 5 sgRNAs (sgCEP55, sgOGG1, sgPTGER2 sgCAPBP7, sgCIT), in RNA-seq but are perturbed with completely different sgRNAs in ATAC-seq (Supplementary Table 4). To understand what makes cluster 0 and 2 different, we performed a differential gene activity test using cells targeted by the top 5 sgRNAs in cluster 0 and 2 ATAC-seq data. We then examined cells targeted by the shared top 5 sgRNAs in cluster 0 and 2 RNA-seq, and we found that the differential genes we identified through ATAC-seq could partially explain the different clustering of these cells in RNA-seq (Fig. 4f). That is, there are distinctive patterns of gene activity between C0 and C2 cells that correlate with distinctive expression of the corresponding genes in these clusters. These distinctive patterns define subsets of cells that cluster separately even though they were targeted with the same sgRNA. Therefore, our integrated representation of these two independent datasets allows us to gain a better understanding of two subpopulations of cells that respond differently to the same gene perturbation.

## Discussion

In this study, we designed an adversarial approach for the integration of single-cell chromatin accessibility and gene expression data. By benchmarking our method against 5 existing integration methods in 5 ATAC-seq/RNA-seq datasets, our showed that sciCAN and Seurat have overall superior performance of data integration. However, sciCAN shows good mutual label transferring either from RNA-seq to ATAC-seq or from ATAC-seq to RNA-seq, while this mutual information is lost via Seurat integration. In cases where researchers may want to translate ATAC-seq to RNA-seq for inferring gene expression, sciCAN would have an advantage over Seurat. We further demonstrated that sciCAN can be applied to different integrative analyses, like co-trajectory, activity-expression velocity, and co-clustering of CRISPR screens. All these results demonstrate that sciCAN could empower integrative single-cell analysis for biological discoveries.

## Methods

### Representation learning

Deep metric learning has shown effective representation learning without supervision. Chen et al. used a simple framework to learn visual representations in a self-supervised manner^50^. They duplicated each image into two counterparts through image perturbation. The goal of learning is to maximize the consistency of any paired replicates in the latent space z. To achieve this goal, NCE is applied as the loss function as shown in (1). In an N-sample batch, there will be *2* *N* samples through data augmentation, and each augmented image *i* has its corresponding counterpart *j* which is the same, despite the added image perturbation. Then, *cos* quantifies the cosine similarity of image *i* and *j/k* in the latent space *z*. Chen et al. demonstrated that this simple framework turns out to be a highly effective way to learn the discriminative representation without supervision. We adapted this approach in our previous study and showed the sample framework can produce discriminative representations for single-cell data^22^. (See this previous SMILE study for in-depth details of how perturbations are added to duplicated data). Because of the property of this metric learning, our method is fully unsupervised. Users do not need to provide cell-type labels to start model training.

1 $$l_{i,j} = - \log \frac{{{{{\mathrm{exp}}}}(cos(z_i,z_j)/\tau )}}{{\mathop {\sum }\nolimits_{k = 1}^{2N} {{{\mathrm{exp}}}}(cos(z_i,z_k)/\tau )}}$$

### Domain adaptation

Generative models with adversarial domain adaptation were successfully shown to transfer targets to source style and have general applications in image translation^51^. Recently, both scGAN^19^ and AD-AE^20^ incorporated adversarial domain adaptation into a generative model for removing batch effects within single-cell expression data. For both studies, the goal is to find a batch-invariant representation for single-cell gene expression data from various sources. To achieve this, they stacked a discriminator to the encoder and trained the discriminator to distinguish which source the cell comes from using the latent space *z* projected by the encoder. Adversarial training, in this case, will push the encoder to approximate the joint distribution and become capable of projecting cells with data from different modalities to the same integrated representation. Here, we also used domain adaptation to train a discriminator to identify the modality source while the encoder is pushed to diminish modality difference.

### Cycle-consistent adversarial network

Besides the use of adversarial domain adaptation above, we further introduced a cycle-consistent adversarial part. This practice stems from a method called cycleGAN, which presented a high-performing outcome for the task of transferring image styles from one domain to another^21^. The success of establishing a connection between two image domains relies on the concept called “cycle consistency”. Starting from the original image, a generator network translates the image to the other domain. Then, a second generator network translates the image back to its original domain. Through this cycle, the translated-back image should be the same as the original image. Based on this information, adversarial training of generators can establish a reversible connection between two image domains. Different from the goal of cycleGAN, we aim to learn joint representation instead of translating chromatin accessibility to gene expression or vice versa. However, the fundamental concept is the same: we establish a cycle from encoder to generator, and from generator back to encoder. Then, the cycle-consistency loss is applied at the level of latent space *z*.

### Data preprocessing

All methods benchmarked in our study require anchoring genes for integration. We used a common practice that transforms the sparse ATAC-seq peak matrix to a gene activity matrix^26,52,53^. Here, we briefly explain the rationale behind this transformation. RNA-seq measures gene expression, so in a matrix of single-cell gene expression data, each row represents one cell, and each column contains expression values of one gene. The whole matrix represents gene expression levels of all genes across all cells. ATAC-seq, on the other hand, quantifies how accessible genomic loci are to regulatory proteins. Therefore, in a matrix of single-cell chromatin accessibility data, each row is one cell (the same as single-cell gene expression data) and each column contains accessibility values of one genomic locus. The sum of accessibility values of all genomic loci upstream of and within one gene body may relate to the potential of transcription of that gene. Therefore, to convert ATAC-seq data to a form that can be compared to RNA-seq data (a matrix of cells by genes), all accessibility peaks upstream of and within each gene body are summed to represent gene activity. In the converted gene activity matrix, each row is one cell, and each column is accessibility values of one gene. Therefore, after conversion, we can do a simple filtering and reordering to match features of chromatin accessibility and gene expression data. The Signac package provides this conversion process, and we ran the code available at https://satijalab.org/signac/articles/pbmc_vignette.html^53^. After we have both a gene activity matrix and a gene expression matrix, we normalize both modality data with (Log + 1)-transformation, which adds 1 as a pseudo count to the matrix before log-transformation. Then, we identify the top 3000 highly variable genes (HVG) for each modality and use all identified HVG as features for integration. To identify the top 3000 HVG, we use Scanpy by calling the highly_variable_genes function^54^.

### Model training

We trained sciCAN in all datasets for 100 epochs. The learning rate starts from 0.005 with 0.0005 weight decay. All weights in the sciCAN model are updated through stochastic gradient descending. In the *NCE* loss function, temperature *τ* is a crucial parameter that affects discriminative power of the final representation. We set as *τ* = 0.15 for the 32-dimension linear-transformed output and *τ* = 0.5 for the 25-dimension SoftMax activated output, which is consistent to the practice in our previous study^22^. Detailed training code is also provided on sciCAN GitHub (https://github.com/rpmccordlab/sciCAN).

### Integration via LIGER

Multimodal single-cell data integration by LIGER was demonstrated in its published tutorial^14^. We used default parameters to perform integration of chromatin accessibility and gene expression data, and the final dimension of integrated representation by LIGER is 20 for all 5 benchmark datasets. Briefly, LIGER uses integrative nonnegative matrix factorization (iNMF) to identify metagenes that are shared between ATAC-seq and RNA-seq^55^. These metagenes are a weighted matrix of factor loadings of observed gene expression/activity. Then, cell loadings of these metagenes are used to perform joint clustering and other downstream analysis. Ideally, representations of cells from both modalities after iNMF should have been integrated in the same latent space and can be visualized via tSNE or UMAP^56,57^.

### Integration via Harmony

Harmony is the second integration method benchmarked in our study. Originally, Harmony was designed to correct batch effects within single-cell RNA-seq datasets^15^. Later, the use of Harmony in multimodal single-cell data integration was discussed in reviews^58,59^. Meanwhile, a batch-correction benchmark study showed that Harmony was ranked among the top 3 methods, with LIGER and Seurat, for integrating single-cell RNA-seq data^23^. Therefore, we included Harmony in our benchmarking of multimodal single-cell data integration. Harmony learns the joint representation through an iterative k-means clustering, and the outcome is a linear correction function that transforms the original principal components (PCs) to the batch-corrected PCs. Batch information is necessary to guide Harmony to distinguish what variation should be diminished during the k-means iterations. Principally, to integrate chromatin accessibility and gene expression data, modality information serves as the same role of batch information. Again, we used the default procedure of Harmony, in which we reduced the whole dataset into the first 30 PCs.

### Integration via Seurat

Seurat uses canonical correlation analysis to learn the shared latent space between two modalities. This approach is different from LIGER, Harmony, and our method, in a way that Seurat will first identify confident cell pairs between the two modalities. Then, Seurat uses these paired cells as anchors to learn a mutual neighborhood graph. Finally, it computes a projection that brings all other cells to this shared latent space. Because of its “anchor” design, Seurat needs pairwise computation of anchor points when datasets come from more than two sources. Since we only deal with the modality difference between chromatin accessibility and gene expression in this study, we do not need to perform pairwise computation of anchor points with Seurat. For benchmarking, we ran Seurat v3 with the tutorial on https://satijalab.org/seurat/archive/v3.0/atacseq_integration_vignette.html, and the final dimension of integrated representation by Seurat would be 50.

### Integration via ArchR

ArchR uses Seurat as infrastructure to integrate RNA-seq with ATAC-seq data. Different from Seurat, ArchR constrains the mapping from ATAC-seq to RNA-seq in a “subspace”. An initial unconstrained mapping was done through Seurat. This step is aimed to estimate what clusters in ATAC-seq have good correspondence to a certain number of clusters in RNA-seq. Then, the “subspace” or constrained mapping will only project a number of clusters in ATAC-seq onto their corresponded clusters in RNA-seq.

### Integration via SMILE

We previously developed SMILE to integrate multimodal single-cell data when cell anchor information was obtained from co-assay profiling. Because sciCAN and SMILE share the same architecture to learn lower dimension hidden space for single-cell data, SMILE also generate 128-dimension hidden space. To use SMILE for integration in this situation, we had to rely on external tool, like Seurat, to identify cell anchors. Once cell anchors identified, SMILE was trained based on anchored data and projected the rest of unanchored data into the joint representation space. A tutorial can be found at SMILE GitHub (https://github.com/rpmccordlab/SMILE).

### Activity-expression velocity

Activity-expression velocity was calculated with scVelo^46^. We replaced the spliced layer with the gene activity matrix and the unspliced layer with the gene expression matrix. To estimate first and second moments, we used the 128-dimension joint space learned by sciCAN, instead of PCA space.

### Data description

We collected unpaired chromatin accessibility and gene expression from 5 studies for benchmarking^28–32^. ATAC-seq and RNA-seq data in data 1 (Cell lines) and data 4 (Mouse skin) have the same number of cells and cell-types. This is because SNARE-seq and SHARE-seq simultaneously profile chromatin accessibility and gene expression features from the same cells^28,31^. Experimentally, each cell in ATAC-seq has its corresponding cell in RNA-seq in SNARE-seq and SHARE-seq data. However, in our study, we blind ourselves to this paired information for all 6 methods. For the other 3 datasets, data from ATAC-seq and RNA-seq were collected separately on different groups of cells. Therefore, they do not necessarily consist of the same number of cells and do not necessarily share the same cellular components. Cell-type annotations were also annotated separately by the authors, and the author-reported annotations serve as ground truth for integration evaluation. We also collected a joint-profiled human PBMC data by 10X Multiome platform to demonstrate that integration by sciCAN preserves biological information. Finally, we collected two independent CRISPR-perturbed single-cell K562 datasets that profiled chromatin accessibility^49^ and gene expression^47^, respectively. The brief description and citations for these 7 datasets are shown in Supplementary Table 1.

### Evaluation

To evaluate integration by each method, we proposed 4 metrics:

#### Modality and cell-type silhouette score

As we mentioned before, sciCAN and SMILE reduce each dataset into 128-dimension spaces, while LIGER reduces the data to 20 dimensions, Harmony to 30, and both Seurat and ArchR to 50. Since the final dimensions of the integrated representations by the 6 methods are not the same, we further used Uniform Manifold Approximation and Projection (UMAP) to reduce them into 2-dimensions with the same UMAP running parameters^60^. Then, we calculated modality and cell-type silhouette scores on the 2D UMAP spaces. A typical silhouette score *S* ranges from −1 to 1. To better reflect the integration outcome, we define modality silhouette as 1-*abs*(*S*) and cell-type silhouette as (1 + *S*)/2. Of note, we used different labels to calculate modality and cell-type silhouette. For modality silhouette, the label used is modality information. A good integration should have chromatin accessibility and gene expression data largely overlapped. Therefore, the absolute value of *S* should be close to zero (regardless of sign), and we then subtract the absolute value of *S* from 1 so that the best score will have a value of 1. For cell-type silhouette, we used the author-reported annotation label to calculate *S* and then scale the output to the range from 0 to 1. Thus, cell-type silhouette of 1 indicates the best integration that preserves cell-type structure.

#### F1 score from RNA-seq to ATAC-seq, and from ATAC-seq to RNA-seq

A useful integration of modalities should have the ability to transfer cell type labels from one datatype to another, either from RNA-seq to ATAC-seq or from ATAC-seq to RNA-seq. Given cell-type label availability from a single modality, the user should be able to predict cell-types for the other modality, with a fair accuracy. To evaluate how friendly the joint representation is for label transferring, we trained a Support Vector Machine (SVM) classifier with one modality and tested it with the other modality. The choice of SVM is simply based on a constant superior performance of SVM classifier across datasets. Then, we used macro F1 and F1 score for each cell type to evaluate SVM classifiers trained with different joint representations by these 6 methods. Macro F1 score is the average of F1 scores for all cell-types, and it can help us reveal if integration is good for non-major cell-types. This is because cell-types are not balanced in most single-cell data and revealing non-major cell-types is critical for most single-cell analysis. A high macro F1 score can suggest that integration is also good for non-major cell-types. Meanwhile, individual F1 scores for all cell type also report which cell-type prediction is the hard case and what is the highest F1 score the classifier can reach to.

### Metric aggregation and ranking

Across all 5 datasets, we first used $S_{cell - type}$, $S_{modality}$, $F1_{RtoA}$, and $F1_{AtoR}$ to compare sciCAN with each of other 5 methods with wilcoxon test. P value lower than 0.01 indicates sciCAN has better performance in that specific metric. To aggregate all metrics and rank performance of integration, we first aggregated modality and cell-type silhouette scores given the calculation $S_{overall} = 0.7 \times S_{cell - type} + 0.3 \times S_{modality}$. This metric aggregation gives more highlight on how well the integration method preserve biological information instead of simply overlapping two modalities. To rank performance of label transferring, we aggregated macro F1 (RtoA) and macro F1 (AtoR) as the calculation $F1_{overall} = 0.5 \times F1_{RtoA} + 0.5 \times F1_{AtoR}$. We expect a good integration should enable a mutual label transferring from both directions. For the overall ranking, we further got the final score $R_{overall} = 0.5 \times S_{overall} + 0.5 \times F1_{overall}$.

## Supplementary information

Supplementary Figures and Tables

## Data Availability

All datasets used in our study are from previously published studies. The data accession in Gene Expression Omnibus or processed data link can be found in Supplementary Table 1.

## Code availability

sciCAN code is provide on GitHub, with its own repository (https://github.com/rpmccordlab/sciCAN).

## References

[1] Macaulay IC, Ponting CP, Voet T (2017). Single-Cell Multiomics: Multiple Measurements from Single Cells. Trends Genet..

[2] Stuart T, Satija R (2019). Integrative single-cell analysis. Nat. Rev. Genet..

[3] Carter B, Zhao K (2021). The epigenetic basis of cellular heterogeneity. Nat. Rev. Genet..

[4] Kelsey G, Stegle O, Reik W (2017). Single-cell epigenomics: Recording the past and predicting the future.. Science..

[5] Wagner DE, Klein AM (2020). Lineage tracing meets single-cell omics: opportunities and challenges. Nat. Rev. Genet..

[6] Ma F, Pellegrini M (2020). ACTINN: automated identification of cell types in single cell RNA sequencing. Bioinforma. (Oxf., Engl.).

[7] Bernstein NJ (2020). Solo: Doublet Identification in Single-Cell RNA-Seq via Semi-Supervised Deep Learning. Cell Syst..

[8] Arisdakessian C, Poirion O, Yunits B, Zhu X, Garmire LX (2019). DeepImpute: an accurate, fast, and scalable deep neural network method to impute single-cell RNA-seq data. Genome Biol..

[9] Lopez R, Regier J, Cole MB, Jordan MI, Yosef N (2018). Deep generative modeling for single-cell transcriptomics. Nat. methods.

[10] Ma A, McDermaid A, Xu J, Chang Y, Ma Q (2020). Integrative Methods and Practical Challenges for Single-Cell Multi-omics. Trends Biotechnol. (Regul. ed.).

[11] Efremova M, Teichmann SA (2020). Computational methods for single-cell omics across modalities. Nat. Methods.

[12] Butler A, Hoffman P, Smibert P, Papalexi E, Satija R (2018). Integrating single-cell transcriptomic data across different conditions, technologies, and species. Nat. Biotechnol..

[13] Hie B, Bryson B, Berger B (2019). Efficient integration of heterogeneous single-cell transcriptomes using Scanorama. Nat. Biotechnol..

[14] Liu J (2020). Jointly defining cell types from multiple single-cell datasets using LIGER. Nat. Protoc..

[15] Korsunsky I (2019). Fast, sensitive and accurate integration of single-cell data with Harmony. Nat. methods.

[16] Polański K (2020). BBKNN: fast batch alignment of single cell transcriptomes. Bioinformatics.

[17] Wang T (2019). BERMUDA: a novel deep transfer learning method for single-cell RNA sequencing batch correction reveals hidden high-resolution cellular subtypes. Genome Biol..

[18] Lotfollahi M, Wolf FA, Theis FJ (2019). scGen predicts single-cell perturbation responses. Nat. methods.

[19] Bahrami, M. et al. Deep feature extraction of single-cell transcriptomes by generative adversarial network. *Bioinformatics (Oxford, England)*, 10.1093/bioinformatics/btaa976 (2020). 10.1093/bioinformatics/btaa976 33226074

[20] Dincer AB, Janizek JD, Lee S-I (2020). Adversarial deconfounding autoencoder for learning robust gene expression embeddings. Bioinforma. (Oxf., Engl.).

[21] Zhu, J.-Y., Park, T., Isola, P. & Efros, A. A. Unpaired Image-to-Image Translation using Cycle-Consistent Adversarial Networks. Preprint at 10.48550/arXiv.1703.10593 (2017).

[22] Xu Y, Das P, McCord RP (2022). SMILE: mutual information learning for integration of single-cell omics data. Bioinformatics.

[23] Tran HTN (2020). A benchmark of batch-effect correction methods for single-cell RNA sequencing data. Genome Biol..

[24] Stuart T (2019). Comprehensive Integration of Single-Cell Data. Cell.

[25] Granja JM (2021). ArchR is a scalable software package for integrative single-cell chromatin accessibility analysis. Nat. Genet..

[26] Wang C (2020). Integrative analyses of single-cell transcriptome and regulome using MAESTRO. Genome Biol..

[27] Pliner HA (2018). Cicero Predicts cis-Regulatory DNA Interactions from Single-Cell Chromatin Accessibility Data. Mol. cell.

[28] Chen S, Lake BB, Zhang K (2019). High-throughput sequencing of the transcriptome and chromatin accessibility in the same cell. Nat. Biotechnol..

[29] Granja JM (2019). Single-cell multiomic analysis identifies regulatory programs in mixed-phenotype acute leukemia. Nat. Biotechnol..

[30] Wang A (2020). Single-cell multiomic profiling of human lungs reveals cell-type-specific and age-dynamic control of SARS-CoV2 host genes. eLife.

[31] Ma S (2020). Chromatin Potential Identified by Shared Single-Cell Profiling of RNA and Chromatin. Cell (Camb.).

[32] Miao Z (2021). Single cell regulatory landscape of the mouse kidney highlights cellular differentiation programs and disease targets. Nat. Commun..

[33] Peng, T., Chen, G. M. & Tan, K. GLUER: integrative analysis of single-cell omics and imaging data by deep neural network. *bioRxiv*, 2021.2001.2025.427845, 10.1101/2021.01.25.427845 (2021).

[34] Lin Y (2022). scJoint integrates atlas-scale single-cell RNA-seq and ATAC-seq data with transfer learning. Nat. Biotechnol..

[35] Jain MS (2021). MultiMAP: dimensionality reduction and integration of multimodal data. Genome Biol..

[36] Corces MR (2016). Lineage-specific and single-cell chromatin accessibility charts human hematopoiesis and leukemia evolution. Nat. Genet..

[37] Velten L (2017). Human haematopoietic stem cell lineage commitment is a continuous process. Nat. Cell Biol..

[38] Buenrostro JD (2018). Integrated Single-Cell Analysis Maps the Continuous Regulatory Landscape of Human Hematopoietic Differentiation. Cell.

[39] Han X (2018). Mapping the Mouse Cell Atlas by Microwell-Seq. Cell.

[40] Rodriguez-Fraticelli AE (2018). Clonal analysis of lineage fate in native haematopoiesis. Nature.

[41] Wolf FA (2019). PAGA: graph abstraction reconciles clustering with trajectory inference through a topology preserving map of single cells. Genome Biol..

[42] La Manno G (2018). RNA velocity of single cells. Nature.

[43] Bergen V, Soldatov RA, Kharchenko PV, Theis FJ (2021). RNA velocity—current challenges and future perspectives. Mol. Syst. Biol..

[44] Xia C, Fan J, Emanuel G, Hao J, Zhuang X (2019). Spatial transcriptome profiling by MERFISH reveals subcellular RNA compartmentalization and cell cycle-dependent gene expression. Proc. Natl Acad. Sci..

[45] Tedesco, M. et al. Chromatin Velocity reveals epigenetic dynamics by single-cell profiling of heterochromatin and euchromatin. *Nature Biotechnology*, 10.1038/s41587-021-01031-1 (2021). 10.1038/s41587-021-01031-1 34635836

[46] Bergen V, Lange M, Peidli S, Wolf FA, Theis FJ (2020). Generalizing RNA velocity to transient cell states through dynamical modeling. Nat. Biotechnol..

[47] Dixit A (2016). Perturb-Seq: Dissecting Molecular Circuits with Scalable Single-Cell RNA Profiling of Pooled Genetic Screens. Cell.

[48] Rubin AJ (2019). Coupled Single-Cell CRISPR Screening and Epigenomic Profiling Reveals Causal Gene Regulatory Networks. Cell.

[49] Pierce SE, Granja JM, Greenleaf WJ (2021). High-throughput single-cell chromatin accessibility CRISPR screens enable unbiased identification of regulatory networks in cancer. Nat. Commun..

[50] Chen, T., Kornblith, S., Norouzi, M. & Hinton, G. In *International conference on machine learning*. 1597–1607 (PMLR, 2020).

[51] Tzeng, E., Hoffman, J., Saenko, K. & Darrell, T. Adversarial Discriminative Domain Adaptation. In *Proceedings of the IEEE conference on computer vision and pattern recognition* pp. 7167–7176 (2017).

[52] Fang R (2021). Comprehensive analysis of single cell ATAC-seq data with SnapATAC. Nat. Commun..

[53] Stuart T, Srivastava A, Madad S, Lareau CA, Satija R (2021). Single-cell chromatin state analysis with Signac. Nat. Methods.

[54] Wolf FA, Angerer P, Theis FJ (2018). SCANPY: large-scale single-cell gene expression data analysis. Genome Biol..

[55] Yang Z, Michailidis G (2016). A non-negative matrix factorization method for detecting modules in heterogeneous omics multi-modal data. Bioinforma. (Oxf., Engl.)..

[56] Kobak D, Berens P (2019). The art of using t-SNE for single-cell transcriptomics. Nat. Commun..

[57] Becht E (2018). Dimensionality reduction for visualizing single-cell data using UMAP. Nat. Biotechnol..

[58] Argelaguet, R., Cuomo, A. S. E., Stegle, O. & Marioni, J. C. Computational principles and challenges in single-cell data integration. *Nat. Biotechnol.*10.1038/s41587-021-00895-7 (2021). 10.1038/s41587-021-00895-7 33941931

[59] Forcato M, Romano O, Bicciato S (2021). Computational methods for the integrative analysis of single-cell data. Brief. Bioinforma..

[60] McInnes, L., Healy, J., Saul, N. & Großberger, L. UMAP: Uniform Manifold Approximation and Projection. *J Open Source Softw.* **3**, 861 (2018).

