# Supplementary material for: sciCAN: single-cell chromatin accessibility and gene expression data integration via cycle-consistent adversarial network
Source: NPJ Syst Biol Appl. 2022 Sep 12;8:33. doi: 10.1038/s41540-022-00245-6 (PMC9464763; doi:10.1038/s41540-022-00245-6)
Supplement: Supplementary file 1 — Supplementary Figures and Tables [file 41540_2022_245_MOESM1_ESM.pdf]

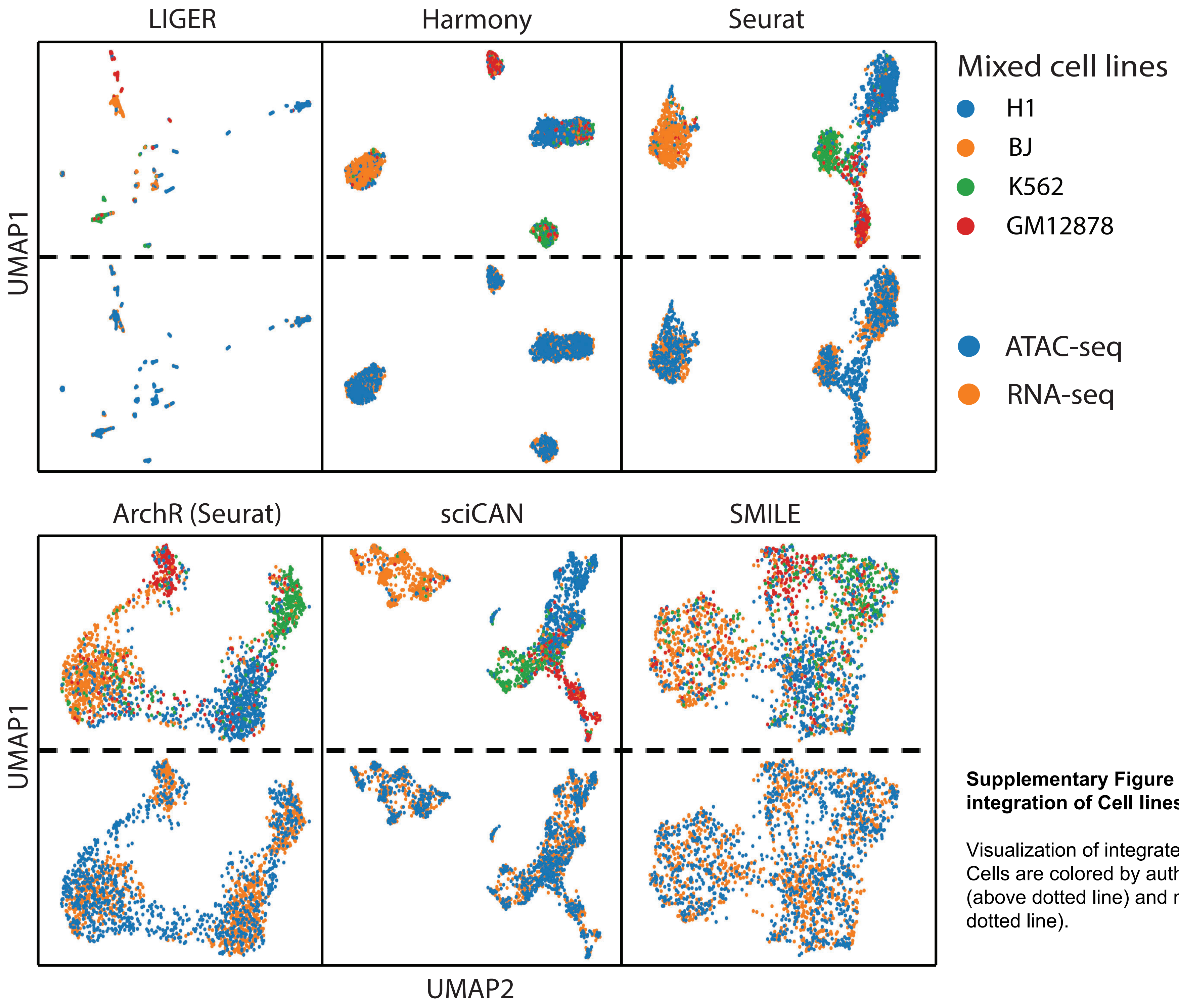

Supplementary Figure 1. Visualization of integration of Cell lines data.

Visualization of integrated cell lines data via UMAP. Cells are colored by author-reported cell types (above dotted line) and modality source (below dotted line).

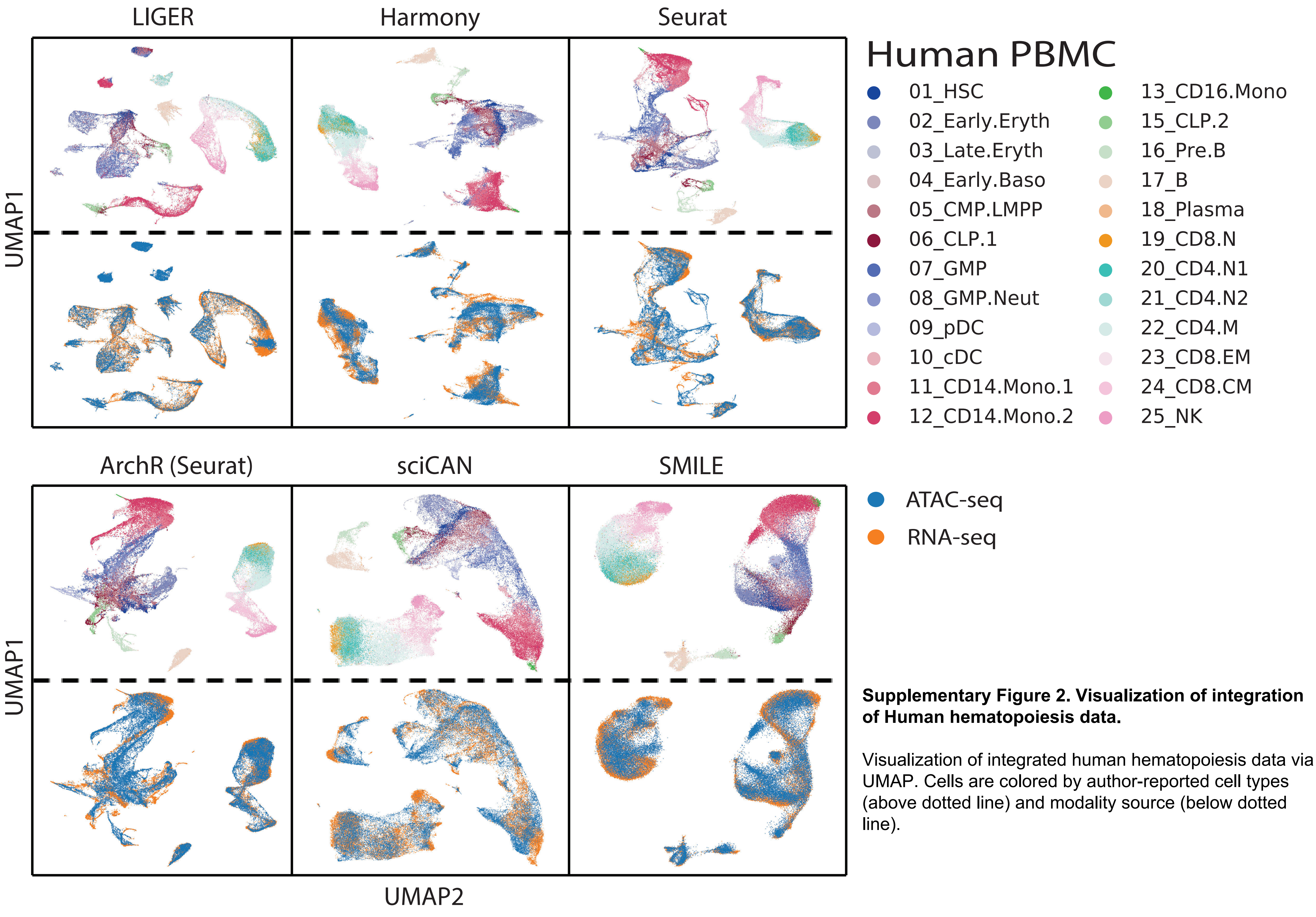

Supplementary Figure 2. Visualization of integration of Human hematopoiesis data.

Visualization of integrated human hematopoiesis data via UMAP. Cells are colored by author-reported cell types (above dotted line) and modality source (below dotted line).

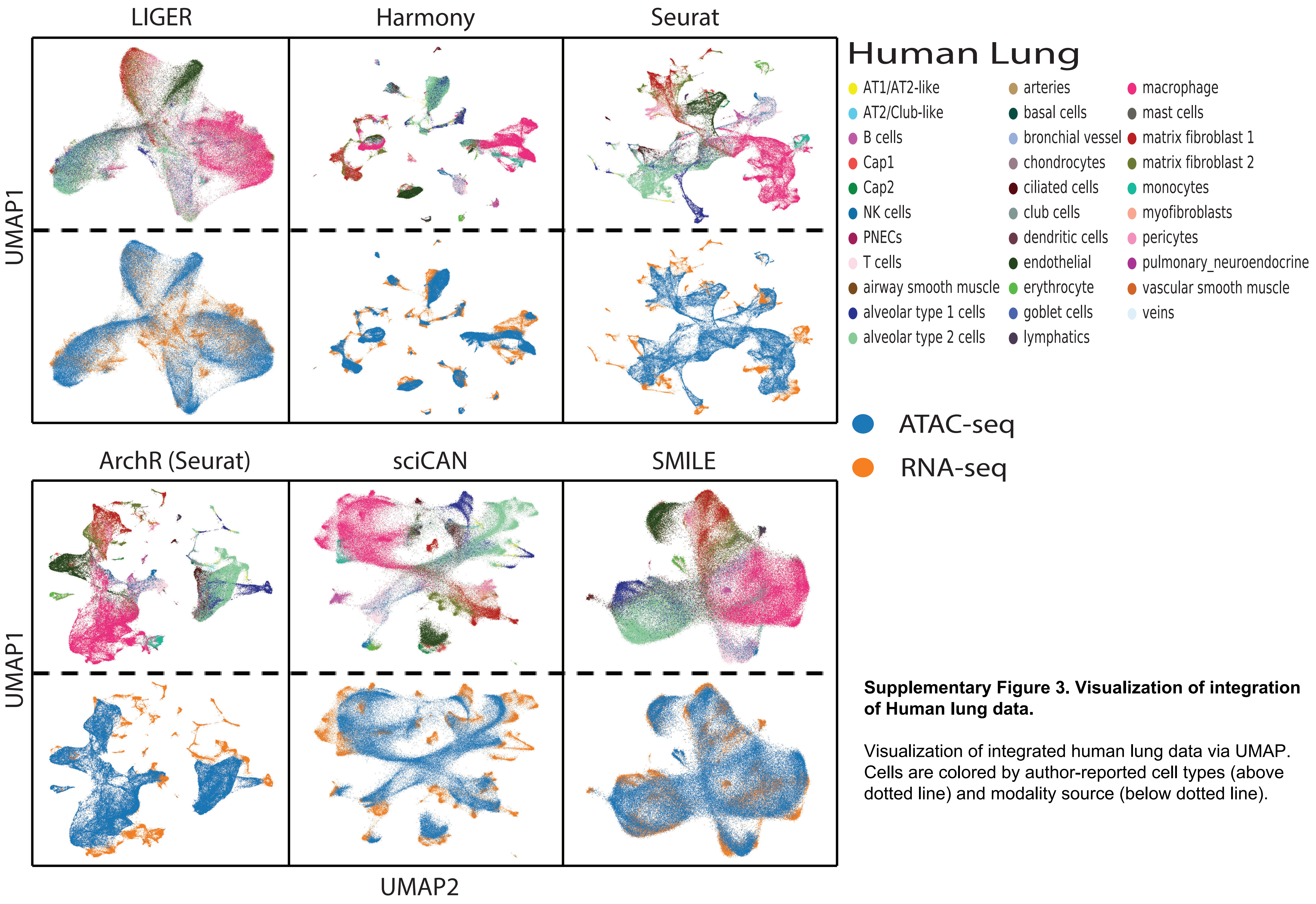

Supplementary Figure 3. Visualization of integration of Human lung data.

Visualization of integrated human lung data via UMAP. Cells are colored by author-reported cell types (above dotted line) and modality source (below dotted line).

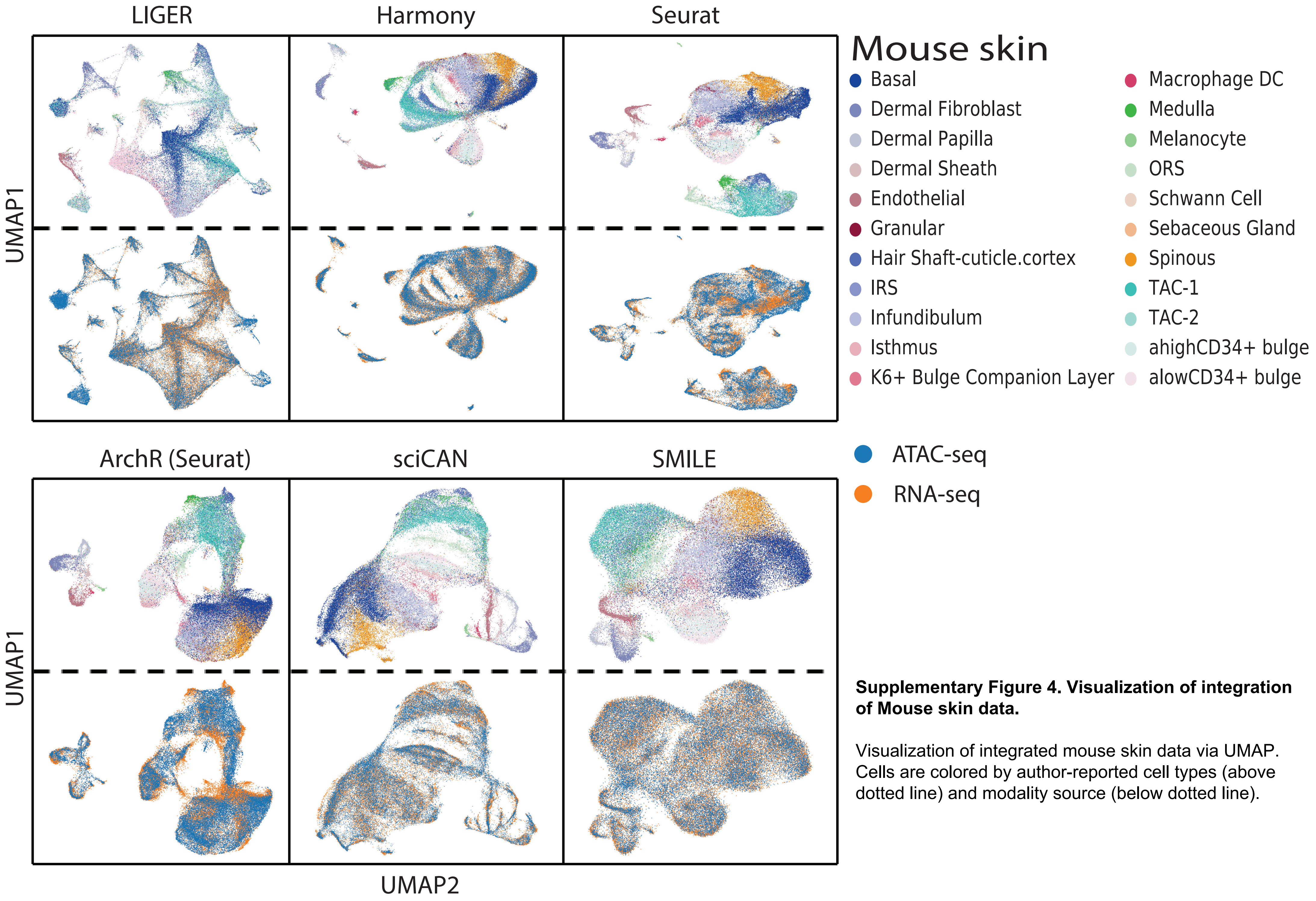

Supplementary Figure 4. Visualization of integration of Mouse skin data.

Visualization of integrated mouse skin data via UMAP. Cells are colored by author-reported cell types (above dotted line) and modality source (below dotted line).

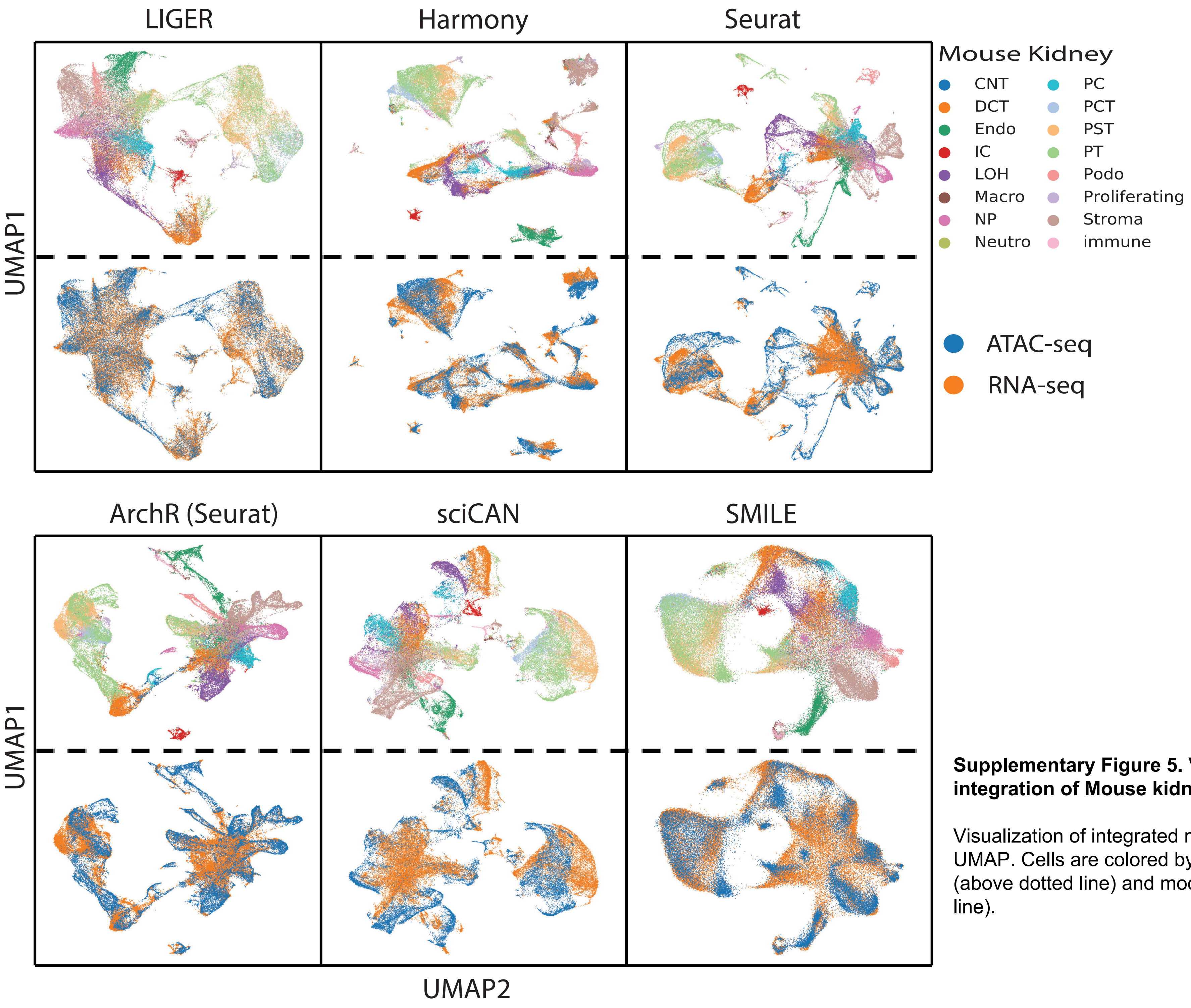

Supplementary Figure 5. Visualization of integration of Mouse kidney data.

Visualization of integrated mouse kidney data via UMAP. Cells are colored by author-reported cell types (above dotted line) and modality source (below dotted line).

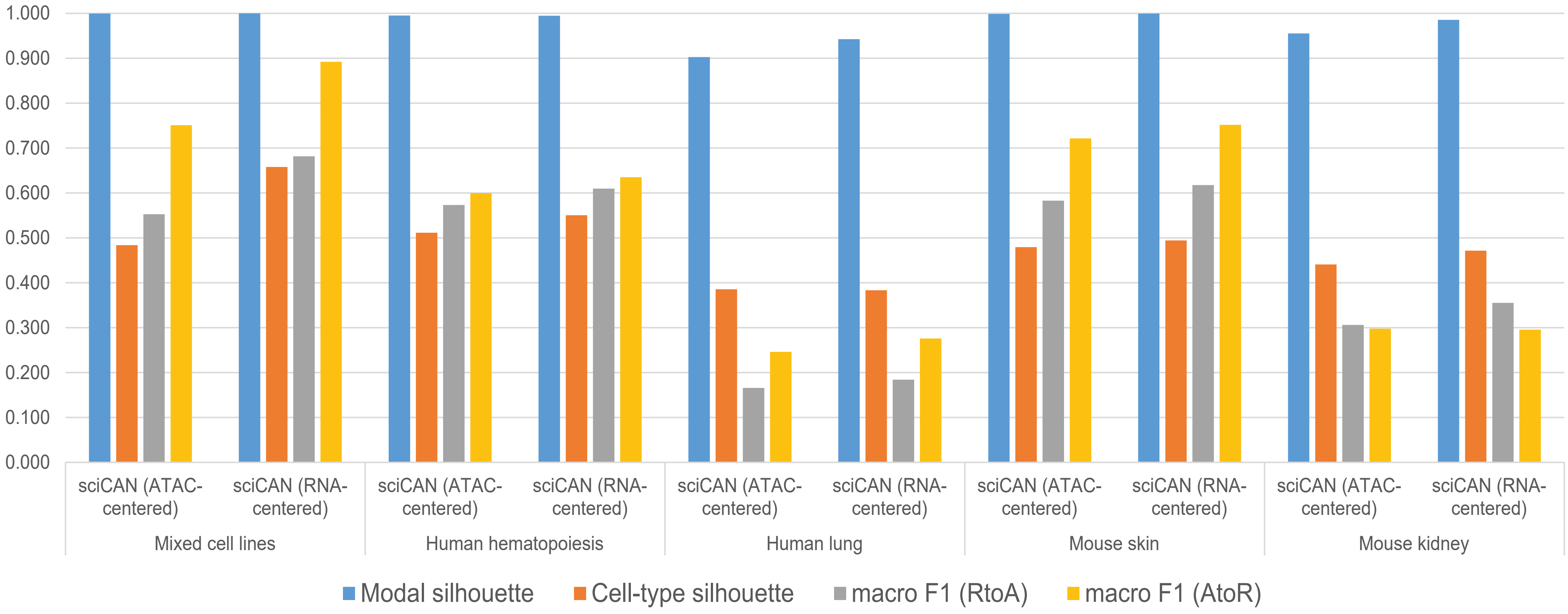

Supplementary Figure 6. Comparison of RNA-centered and ATAC-centered integration by sciCAN.

Performances of RNA-centered and ATAC-centered sciCAN were evaluated by modality- and cell-type silhouette scores, and RtoA and AtoR macro F1 scores where R = RNA-seq and A = ATAC-seq.

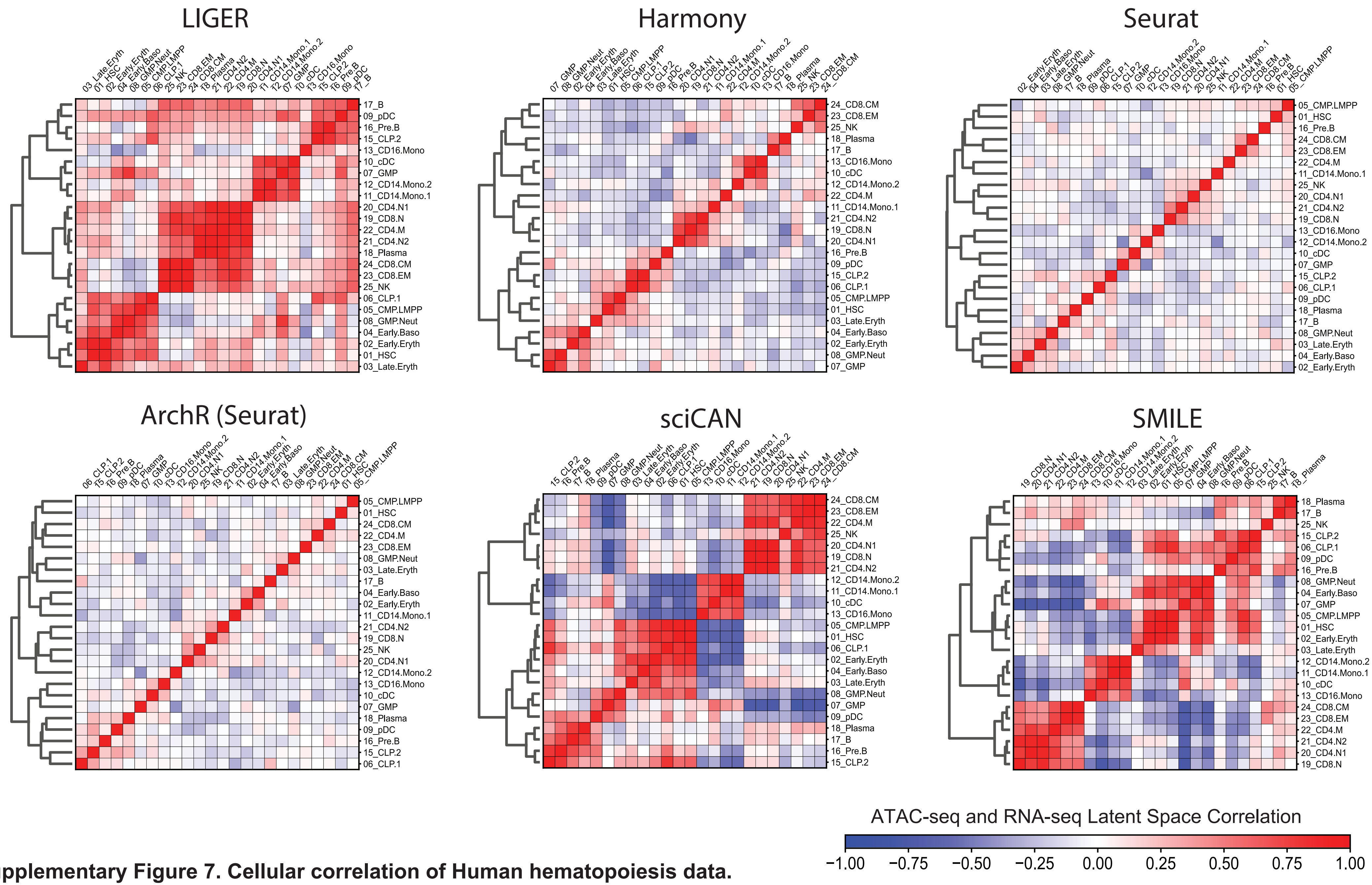

Supplementary Figure 7. Cellular correlation of Human hematopoiesis data.
Cellular correlation based on integrated latent space. After integration, cells in the same cell type from both RNA-seq and ATAC-seq modalities were aggregated into one sample. Author-reported cell-type information was used for aggregation. Spearman correlation was then calculated using aggregated latent spaces returned by different integration methods. We note that the sciCAN latent space allows us to detect strong clusters of related cell types while other methods that score high on most benchmarks (Seurat and Harmony) report weaker correlations that sometimes separate cell types known to be related (CD14.Mono.1 and CD14.Mono.2 for example).

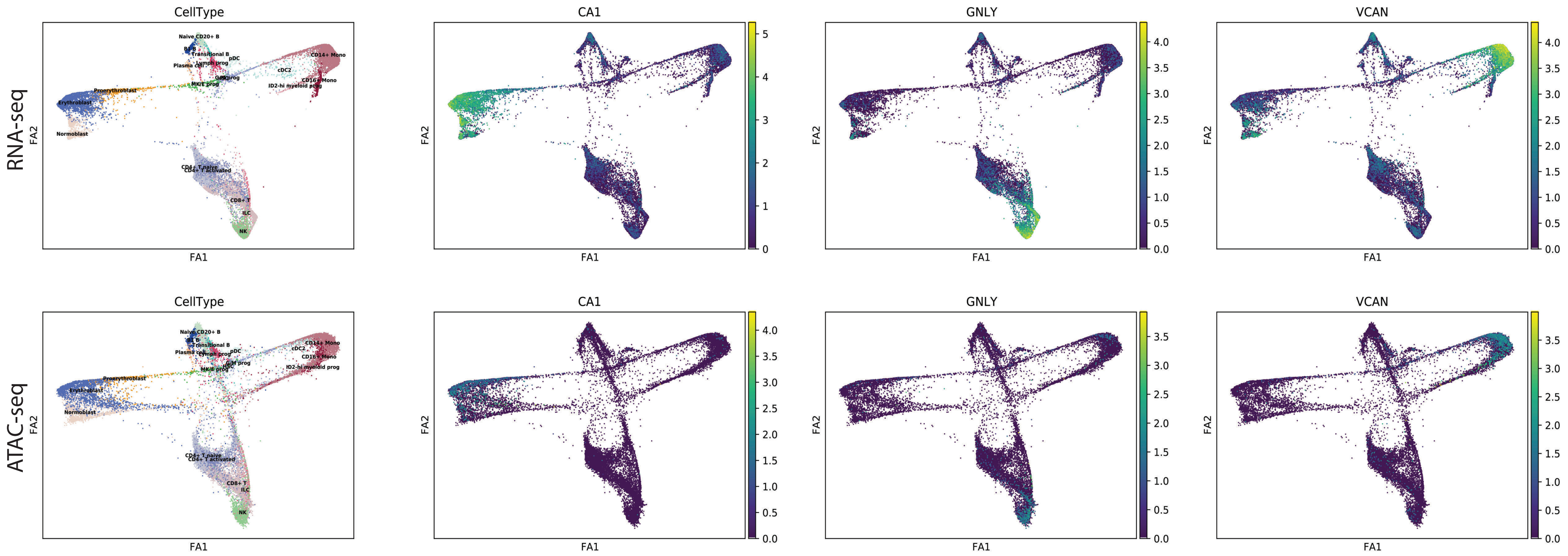

Supplementary Figure 8. Visualization of joint trajectory of hematopoiesis constructed through PAGA.
Co-trajectory visualization without aggregating cells to one cell-type dot. In leftmost panel, colors correspond to labeled cell types. In right panels, color stands for the expression (top) or gene activity (bottom) of the gene indicated above each graph.

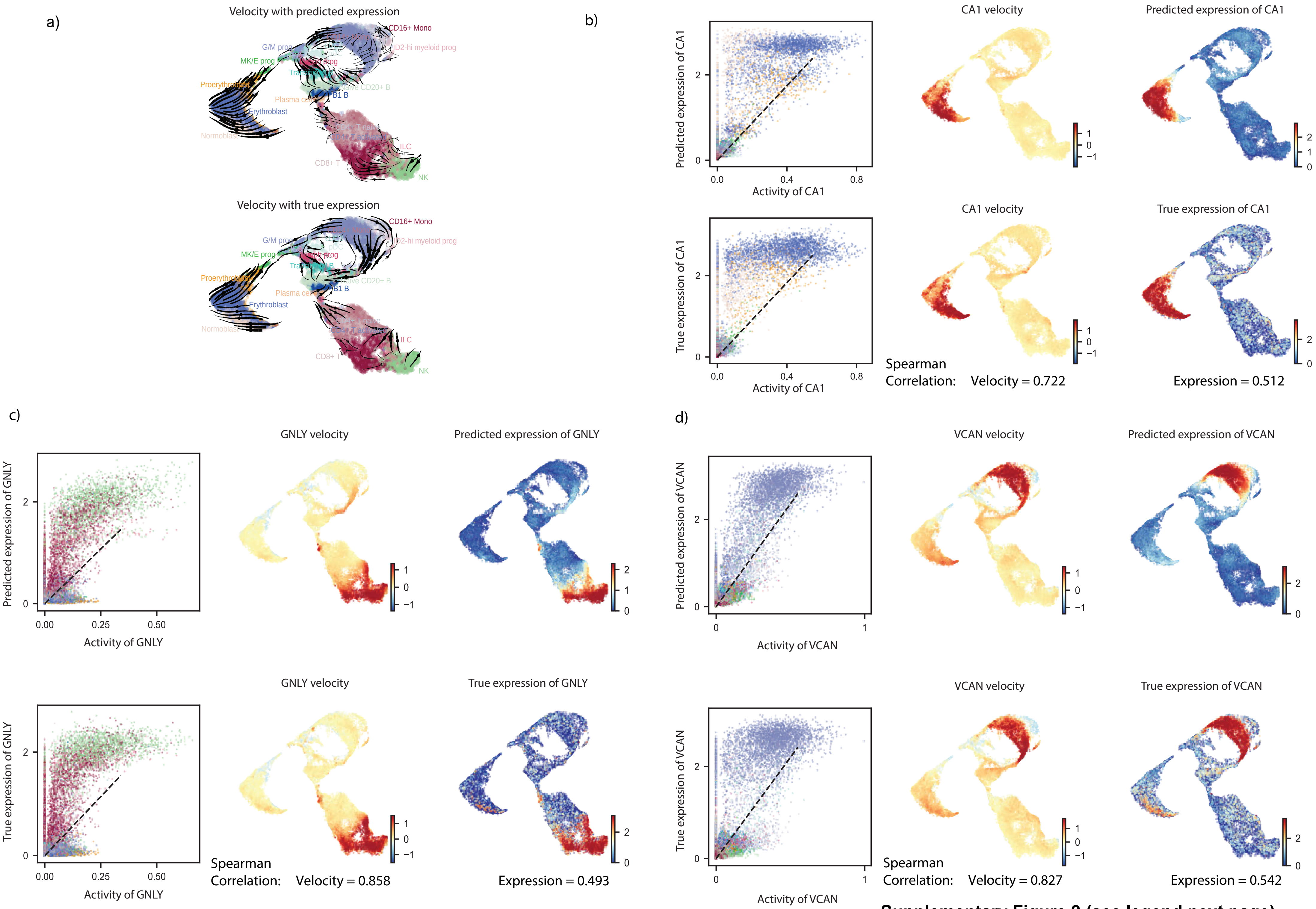

Supplementary Figure 9 (see legend next page)

Supplementary Figure 9. Activity-expression velocity of the hematopoietic hierarchy.

a) Activity-expression velocity was calculated using predicted expression data (upper panel) or true expression data (lower panel) and displayed on the single cell representation as arrows indicating the magnitude and direction of the flow from higher gene activity to higher gene expression across single cells. Cells are colored according to labeled cell types. b) Activity-expression velocity of signature gene CA1 based on either predicted expression data (upper panels) or true expression data (lower panels). Left: CA1 expression (predicted or measured) is plotted vs. gene activity (accessibility) for each cell. Cell type indicated by color that corresponds to labels in previous panels. Dotted line indicates an estimated 'steady-state' ratio. The area above the dotted line suggests positive velocity, in which increased gene expression precedes increased gene accessibility. Middle: the calculated velocity of CA1 superimposed onto the integrated representation across the hematopoietic hierarchy. Right: the expression of CA1 predicted by ATAC-seq

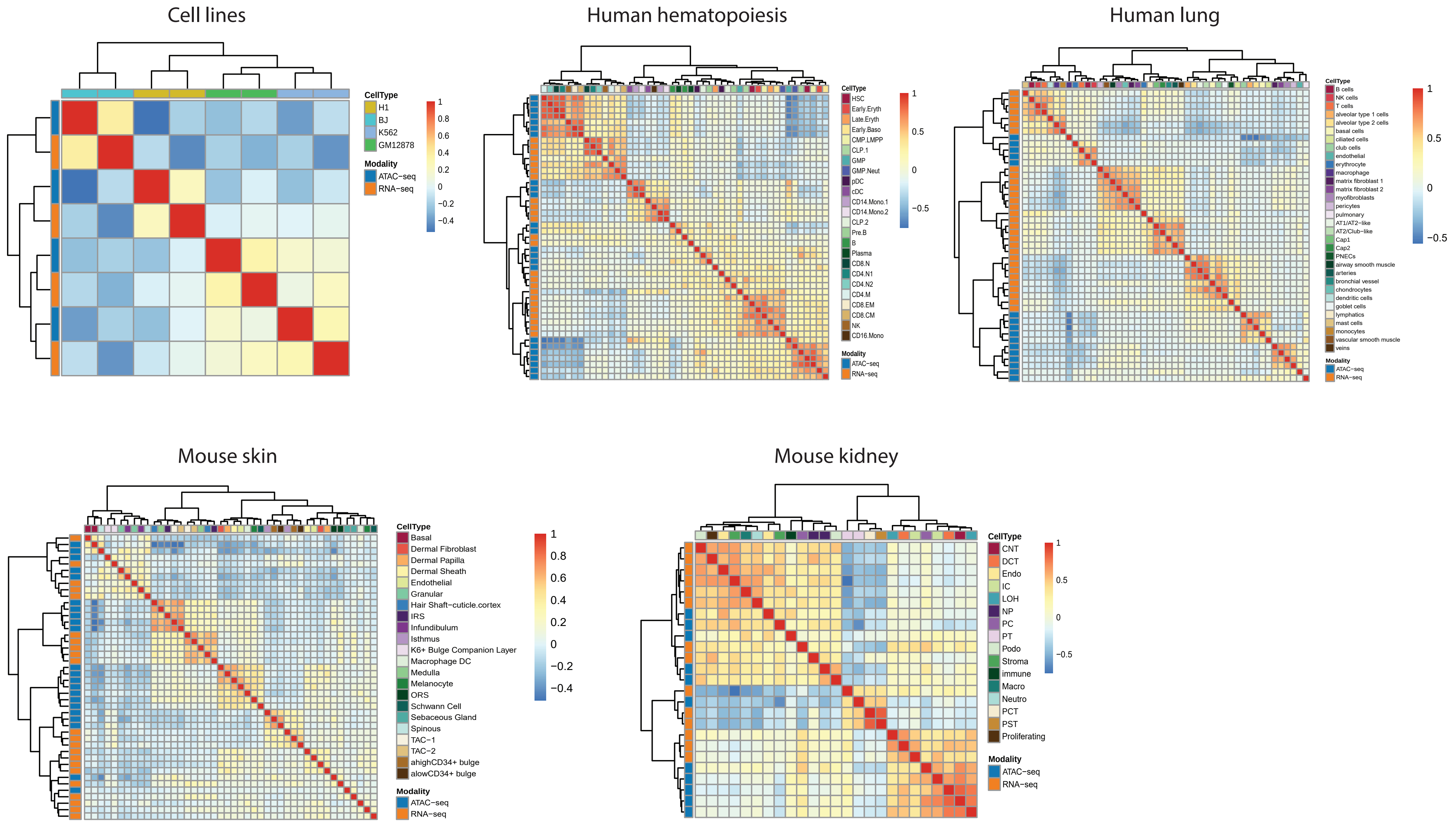

Supplementary Figure 10. Spearman correlations of cell types in 5 benchmark data.
Spearman correlation between RNA-seq and ATAC-seq profiles of cells in different cell types in both modalities. Gene expression or gene activity matrix was averaged by author-reported cell types.

Data	Sample	# of cells in ATAC-seq	# of cells in RNA-seq	# of cell-types in ATAC-seq	# of cell-types in RNA-seq	Accession	Reference	Processed data
1	Cell lines	1047	1047	4	4	GSE126074	Chen et al., 2019	
2	Human hematopoiesis	33819	34901	23	24	GSE139369	Granja et al., 2019	
3	Human lung	82159	44294	16	30	GSE161383	Wang et al., 2020	
4	Mouse skin	32231	32231	22	22	GSE140203	Ma et al., 2020	
5	Mouse kidney	28316	43410	11	14	GSE157079	Miao et al., 2021	
6	Human PBMC	20952	20952	21	21	NA	NA	https://support.10xgenomics.com/single-cell-multiome-atac-gex/datasets
7	K562	9797	22656	NA	NA	GSE90063/ GSE168851	Dixit et al., 2016/ Pierce et al., 2021	

Supplementary Table 1. Dataset descriptions.

All datasets used in this study are public. The number of available cells and annotated cell types are briefly described. The corresponding GEO accessions and links of processed data are also provided in this table.

	LIGER	Harmony	Seurat	ArchR	SMILE
**Cell type sillhouette**	Yes	No	No	Yes	Yes
**Modality sillhouette**	Yes	No	Yes	Yes	No
**F1 (RtoA)**	Yes	Yes	No	Yes	Yes
**F1 (AtoR)**	Yes	Yes	Yes	Yes	Yes

Supplementary Table 2. Statistical test for method comparison.
Benchmarking metrics as plotted in Figure 2a and b were used for statistical comparisons of each method to sciCAN. The scores for each method on each subsampled dataset were paired with the sciCAN results and compared using a one-sided matched pair Wilcoxon signed rank test. Comparisons labeled "Yes" yielded a p-value <0.01 when testing the alternative hypothesis that sciCAN scored lower than the other method. Thus "Yes" indicates that sciCAN score significantly higher than the indicated method for the indicated metric.

Model performance (PBMC 10X Mulitome)				Loss parameter set				
Cell-type silhouette	Modal silhouette	F1 (RtoA)	F1 (AtoR)	L_{NCErna}	$L_{NCEatac}$	L_{rna}	$L_{atac}+L_{cyc}$	
0.558	0.9974	0.577	0.746	2.00	0.50	1.50	5.00	(parameters used for main results)
0.547	0.9981	0.540	0.726	1.00	1.00	1.00	1.00	
0.549	0.9974	0.552	0.730	1.00	1.00	1.00	3.00	
0.543	0.9988	0.523	0.712	1.00	1.00	1.00	5.00	
0.548	0.9960	0.520	0.708	1.00	1.00	3.00	1.00	
0.549	0.9975	0.531	0.724	1.00	1.00	5.00	1.00	
0.560	0.9963	0.562	0.738	2.00	1.00	1.00	3.00	
0.566	0.9978	0.552	0.724	2.00	1.00	3.00	1.00	
0.570	0.9962	0.554	0.736	2.00	1.00	3.00	3.00	
0.524	0.9969	0.493	0.661	0.50	1.00	1.00	3.00	
0.558	0.9953	0.530	0.691	0.50	1.00	3.00	1.00	
0.547	0.9977	0.525	0.688	0.50	1.00	3.00	3.00	
0.547	0.9963	0.492	0.663	1.00	2.00	1.00	3.00	
0.549	0.9971	0.492	0.677	1.00	2.00	3.00	1.00	
0.546	0.9973	0.506	0.695	1.00	2.00	3.00	3.00	
0.559	0.9972	0.547	0.722	1.00	0.50	1.00	3.00	
0.565	0.9970	0.553	0.732	1.00	0.50	3.00	1.00	
0.559	0.9980	0.548	0.711	1.00	0.50	3.00	3.00	

Model performance (Mixed cell lines SNARE-seq)				Loss parameter set				
Cell-type silhouette	Modal silhouette	F1 (RtoA)	F1 (AtoR)	L_{NCErna}	$L_{NCEatac}$	L_{rna}	$L_{atac}+L_{cyc}$	
0.639	0.9922	0.645	0.920	2.00	0.50	1.50	5.00	(parameters used for main results)
0.609	0.9966	0.597	0.903	1.00	1.00	1.00	1.00	
0.614	0.9954	0.599	0.897	1.00	1.00	1.00	3.00	
0.611	0.9953	0.589	0.898	1.00	1.00	1.00	5.00	
0.598	0.9988	0.593	0.837	1.00	1.00	3.00	1.00	
0.595	0.9997	0.594	0.824	1.00	1.00	5.00	1.00	
0.633	0.9934	0.634	0.912	2.00	1.00	1.00	3.00	
0.611	0.9974	0.625	0.845	2.00	1.00	3.00	1.00	
0.625	0.9975	0.630	0.843	2.00	1.00	3.00	3.00	
0.588	0.9948	0.559	0.879	0.50	1.00	1.00	3.00	
0.564	0.9992	0.548	0.815	0.50	1.00	3.00	1.00	
0.534	0.9999	0.554	0.793	0.50	1.00	3.00	3.00	
0.581	0.9983	0.548	0.863	1.00	2.00	1.00	3.00	
0.554	0.9997	0.550	0.794	1.00	2.00	3.00	1.00	
0.541	0.9998	0.539	0.771	1.00	2.00	3.00	3.00	
0.638	0.9922	0.616	0.929	1.00	0.50	1.00	3.00	
0.635	0.9979	0.638	0.870	1.00	0.50	3.00	1.00	
0.633	0.9979	0.632	0.846	1.00	0.50	3.00	3.00	

Supplementary Table 3. Evaluating coefficients of loss functions.

Evaluation of integration in two datasets with different coefficients for each part of the whole loss function. Integration outcomes are evaluated by cell-type silhouette, modality silhouette, macro F1 (RtoA), and macro F1 (AtoR).

RNA-seq

C0

Rank	Cluster	# cells	Proportion
1	sgCEP55	1727	0.1401
2	sgOGG1	1667	0.1352
3	sgPTGER2	1637	0.1328
4	sgCABP7	1154	0.0936
5	sgCIT	1141	0.0926
6	sgARHGEF17	934	0.0758
7	sgCENPE	841	0.0682
8	sgAURKC	836	0.0678
9	sgECT2	651	0.0528
10	sgELK1	458	0.0372
11	sgAURKB	380	0.0308
12	sgRACGAP1	370	0.0300
13	sgTOR1AIP1	281	0.0228
14	sgAURKA	188	0.0153
15	**sgELF1**	16	**0.0013**
16	sgEGR1	13	0.0011
17	sgE2F4	7	0.0006
18	sgNR2C2	6	0.0005
19	**sgYY1**	6	**0.0005**
20	**sgGABPA**	5	**0.0004**
21	sgCREB1	4	0.0003
22	sgIRF1	4	0.0003
23	sgETS1	1	0.0001
	SUM	12327	

C1

Rank	Cluster	# cells	Proportion
1	sgELK1	1615	0.1962
2	**sgELF1**	1296	**0.1574**
3	sgCREB1	882	0.1071
4	sgEGR1	787	0.0956
5	sgETS1	641	0.0779
6	**sgYY1**	604	**0.0734**
7	**sgGABPA**	573	**0.0696**
8	sgNR2C2	565	0.0686
9	sgE2F4	491	0.0596
10	sgIRF1	490	0.0595
11	sgPTGER2	45	0.0055
12	sgCEP55	41	0.0050
13	sgOGG1	39	0.0047
14	sgCABP7	37	0.0045
15	sgARHGEF17	24	0.0029
16	sgCENPE	18	0.0022
17	sgCIT	18	0.0022
18	sgAURKC	15	0.0018
19	sgECT2	13	0.0016
20	sgTOR1AIP1	12	0.0015
21	sgAURKA	11	0.0013
22	sgAURKB	8	0.0010
23	sgRACGAP1	8	0.0010
	SUM	8233	

C2

Rank	Cluster	# cells	Proportion
1	sgCEP55	300	0.1431
2	sgPTGER2	291	0.1388
3	sgOGG1	251	0.1198
4	sgCABP7	203	0.0969
5	sgCIT	201	0.0959
6	sgARHGEF17	153	0.0730
7	sgCENPE	152	0.0725
8	sgAURKC	149	0.0711
9	sgECT2	110	0.0525
10	sgELK1	81	0.0386
11	sgRACGAP1	68	0.0324
12	sgAURKB	56	0.0267
13	sgTOR1AIP1	51	0.0243
14	sgAURKA	28	0.0134
15	**sgELF1**	1	**0.0005**
16	sgNR2C2	1	0.0005
17	sgCREB1	0	0.0000
18	sgE2F4	0	0.0000
19	sgEGR1	0	0.0000
20	sgETS1	0	0.0000
21	**sgGABPA**	0	**0.0000**
22	sgIRF1	0	0.0000
23	**sgYY1**	0	**0.0000**
	SUM	2096	

ATAC-seq

C0

Rank	Cluster	# cells	Proportion
1	sgFOSL1	193	0.0368
2	sgSETDB1	187	0.0357
3	**sgELF1**	164	**0.0313**
4	sgCEBPZ	163	0.0311
5	sgPBX2	162	0.0309
6	sgZBTB11	162	0.0309
7	sgATF1	161	0.0307
8	sgTRIM28	161	0.0307
9	sgCUX1	160	0.0305
10	sgBCLAF1	159	0.0303
11	sgTBP	159	0.0303
12	sgCEBPB	157	0.0299
13	sgBRF2	155	0.0296
14	sgHINFP	152	0.0290
15	sgRPL9	152	0.0290
16	sgZNF280A	152	0.0290
17	sgNFYB	151	0.0288
18	sgNFE2	150	0.0286
19	sgZNF407	142	0.0271
20	sgTFDP1	140	0.0267
21	sgPOLR1D	139	0.0265
22	**sgGABPA**	136	**0.0259**
23	sgREST	134	0.0255
24	**sgYY1**	134	**0.0255**
25	sgZZZ3	133	0.0254
26	sgARID2	130	0.0248
27	sgCTCF	120	0.0229
28	sgTHAP1	120	0.0229
29	sgNRF1	119	0.0227
30	sgHSPA5	109	0.0208
31	sgMAX	105	0.0200
32	sgKLF16	95	0.0181
33	sgATF3	92	0.0175
34	sgMYC	88	0.0168
35	sgARID3A	87	0.0166
36	sgGTF2B	76	0.0145
37	sgCDC5L	75	0.0143
38	sgKLF1	66	0.0126
39	sgCAD	61	0.0116
40	sgGATA1	44	0.0084
	SUM	5245	

C1

Rank	Cluster	# cells	Proportion
1	sgZNF280A	122	0.0384
2	sgTFDP1	113	0.0355
3	**sgELF1**	111	**0.0349**
4	sgNFYB	107	0.0336
5	sgATF1	103	0.0324
6	sgHINFP	103	0.0324
7	sgCEBPZ	100	0.0314
8	sgCUX1	100	0.0314
9	sgZNF407	99	0.0311
10	sgBCLAF1	97	0.0305
11	sgZBTB11	96	0.0302
12	sgBRF2	94	0.0296
13	sgPBX2	94	0.0296
14	sgTBP	93	0.0292
15	sgTRIM28	90	0.0283
16	sgREST	89	0.0280
17	sgCEBPB	87	0.0273
18	sgSETDB1	85	0.0267
19	sgZZZ3	82	0.0258
20	sgFOSL1	80	0.0251
21	sgNRF1	80	0.0251
22	sgARID2	78	0.0245
23	sgNFE2	74	0.0233
24	sgKLF16	72	0.0226
25	sgCAD	71	0.0223
26	sgCDC5L	71	0.0223
27	sgMAX	71	0.0223
28	**sgYY1**	70	**0.0220**
29	sgCTCF	69	0.0217
30	sgPOLR1D	67	0.0211
31	sgHSPA5	64	0.0201
32	sgARID3A	59	0.0185
33	sgKLF1	59	0.0185
34	sgGTF2B	54	0.0170
35	sgGATA1	52	0.0163
36	sgMYC	51	0.0160
37	**sgGABPA**	49	**0.0154**
38	sgTHAP1	49	0.0154
39	sgRPL9	39	0.0123
40	sgATF3	37	0.0116
	SUM	3181	

C2

Rank	Cluster	# cells	Proportion
1	sgTRIM28	58	0.0423
2	sgGATA1	50	0.0365
3	sgATF1	49	0.0357
4	sgZNF280A	48	0.0350
5	sgZNF407	47	0.0343
6	sgPBX2	42	0.0306
7	sgCEBPZ	41	0.0299
8	sgMAX	41	0.0299
9	sgTFDP1	41	0.0299
10	sgFOSL1	40	0.0292
11	**sgELF1**	39	**0.0284**
12	**sgGABPA**	39	**0.0284**
13	sgSETDB1	39	0.0284
14	sgTBP	39	0.0284
15	sgZZZ3	39	0.0284
16	sgNRF1	38	0.0277
17	sgZBTB11	38	0.0277
18	sgCUX1	37	0.0270
19	sgBRF2	36	0.0263
20	sgCEBPB	34	0.0248
21	sgHSPA5	34	0.0248
22	sgKLF1	34	0.0248
23	sgNFE2	34	0.0248
24	sgNFYB	34	0.0248
25	sgREST	33	0.0241
26	sgPOLR1D	31	0.0226
27	sgARID2	30	0.0219
28	sgBCLAF1	28	0.0204
29	sgCDC5L	28	0.0204
30	sgTHAP1	28	0.0204
31	sgHINFP	27	0.0197
32	sgCTCF	24	0.0175
33	**sgYY1**	24	**0.0175**
34	sgATF3	23	0.0168
35	sgGTF2B	22	0.0160
36	sgKLF16	22	0.0160
37	sgRPL9	22	0.0160
38	sgCAD	21	0.0153
39	sgARID3A	20	0.0146
40	sgMYC	17	0.0124
	SUM	1371	

Supplementary Table 4. Ranking of sgRNAs in each cluster in both RNA-seq and ATAC-seq data.

sgRNAs were ordered from the most to the least represented in each identified cluster for both RNA-seq and ATAC-seq from K562 CRISPR screen data.
